# Surgery combined with radio-chemotherapy for esophageal mucoepidermoid carcinoma: A case report

**DOI:** 10.1097/MD.0000000000011165

**Published:** 2018-06-15

**Authors:** Chunhui Zheng, Xiaomei Chen, Fangbiao Zhang, Liping Yan, Xiangyan Zhang

**Affiliations:** a Department of Cardiothoracic Surgery; b Operating Room; c Department of Pathology, Lishui Hospital of Zhejiang University, Lishui Central Hospital, Lishui, Zhejiang Province, P.R. China.

**Keywords:** esophagus, primary mucoepidermoid carcinoma, radio-chemotherapy, surgery

## Abstract

**Rationale:**

Primary mucoepidermoid carcinoma (MEC) of the esophagus is a rare type of malignant neoplasm. Its morphology resembles that of MEC of the salivary glands. It is characterized by a diffuse mixture of squamous and mucus-secreting glandular carcinoma cells. Due to the low incidence of esophageal MEC, the biological behavior and treatment of this tumor have not been well studied.

**Patient concerns:**

In this case report, we describe a case of a 59-year-old man who presented with difficulty in swallowing. Iohexol swallowing revealed a malignant-appearing structure in the inferior-thoracic region.

**Diagnoses:**

Biopsy of the lesion under endoscopy demonstrated a mucoepidermoid carcinoma of the esophagus.

**Interventions:**

We performed esophagectomy, esophagogastrostomy through the esophageal bed and 2-field lymphadenectomy. Histopathological analysis of the tumor revealed histological characteristics typical of an esophageal MEC. Radio-chemotherapy was administered to this patient.

**Outcomes:**

Seventeen months after surgery, an esophageal computed tomography (CT) scan revealed that the wall of esophagus was evenly thickened. However, endoscopic assessment revealed no evidence of recurrence. Further CT scans at 19 and 31 months after surgery also showed a thickened esophageal wall, although endoscopic assessment at 31 months still revealed no esophageal stricture and no evidence of recurrence. The patient is alive with no dysphagia and no evidence of recurrence for over 39 months.

**Lessons:**

There is little evidence of effective treatment nor guidelines for treatment of esophageal MEC. Although the general prognosis of esophageal MEC is poor, comprehensive treatment of surgery and radio-chemotherapy appeared to be effective in this case. Radio-chemotherapy is a possible treatment option that was shown to have acceptable short-term effects.

## 1 Introduction

Mucoepidermoid carcinoma (MEC) is the most common malignant neoplasm in the salivary glands, sometimes arising in the lacrimal and tracheobronchial glands, and rarely presenting in the esophagus. It is commonly characterized by the presence of epidermoid squamous cells, mucus-secreting cells, and intermediate cells and is histologically diagnosed by Periodic Acid-Schiff staining. Primary esophageal MEC accounts for between 0.05% and 2.2% of all cases of primary esophageal cancer.^[1]^ Its biological behavior and response to therapy have not been well studied, and the standard treatment regimen for esophageal MEC is deficiency. Surgery is a primary treatment option for patients with esophageal MEC who have resectable lesions, but the prognosis is poor. We report here a case of esophageal MEC which was treated with surgical resection and radio-chemotherapy.

### 1.1 Patient consent

The patient provided informed consent for publication of this case report and any associated images. This case report was reviewed and approved by the Clinical College, Lishui Central Hospital Institutional Review Board.

## 2 Case report

### 2.1 History and presentation

A 59-year-old man presented to our institution in September 2014 with a 2-month history of progressive dysphagia, without hoarseness. He had smoked 60 cigarettes per day for 40 years and had been a heavy alcohol drinker for 40 years. For the last 9 years, he had had hypertension, and had been diagnosed with type 2 diabetes over 1 year ago. He had no prior malignant disease or distant metastases.

### 2.2 Examination

**Figure 1:**
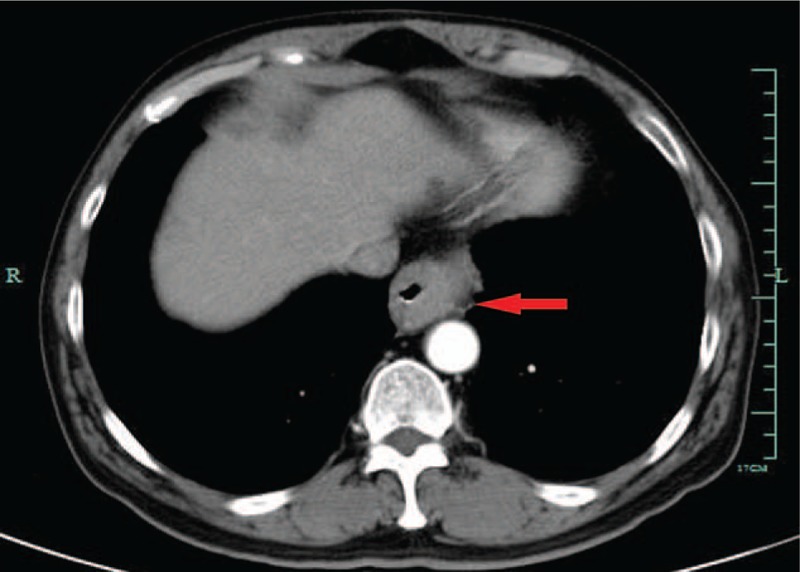
The tumor formed a luminal stenosis and was deemed to be resectable after a CT scan (arrow). CT = computed tomography.

On physical examination, the neck and supraclavicular lymph nodes were not palpable. No significant abnormal values were detected in the blood count or in serum and biochemical analysis. Iohexol swallowing revealed a malignant-appearing structure in the inferior-thoracic region. Endoscopic assessment revealed a lesion of mucosal hyperplasia forming a luminal stenosis 36 cm from the incisor teeth, which rendered it difficult for the gastroscope to pass through it. Biopsy of the lesion demonstrated an esophageal MEC. On computed tomography (CT) scanning, the tumor was deemed to be resectable (Fig. 1). As the patient was found to be medically fit for an esophageal resection, this surgery was performed.

### 2.3 Surgery

Esophagectomy, 2-field (upper abdominal and mediastinum) lymphadenectomy, and esophagogastrostomy through the esophageal bed were performed on the tenth day of hospital admission. The resected esophagectomy specimen contained a fungating tumor that measured 45 and 40 mm in the longitudinal and cross-sectional dimensions.

### 2.4 Pathological findings

**Figure 2:**
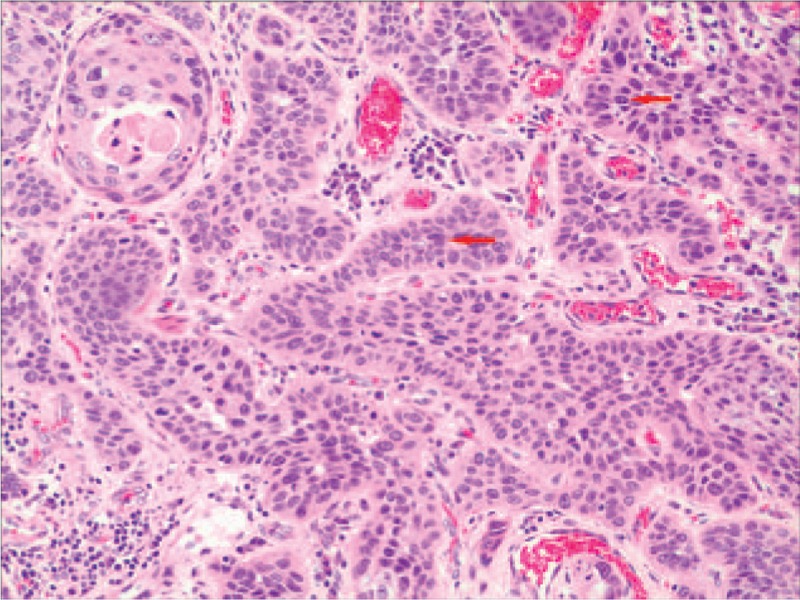
The tumor was composed of epithelioid cells, distribution of nests (arrow) (HE staining; magnification, 100×). HE = hematoxylin and eosin.

**Figure 3:**
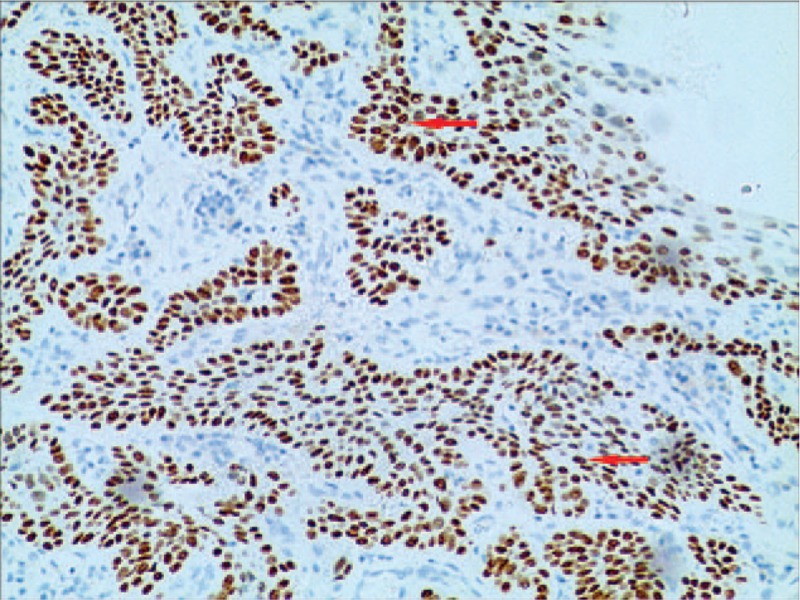
Section of tumor tissue stained using the EnVision method, and the P-63 was positive (arrow) (EnVision method; magnification, 200×).

**Figure 4:**
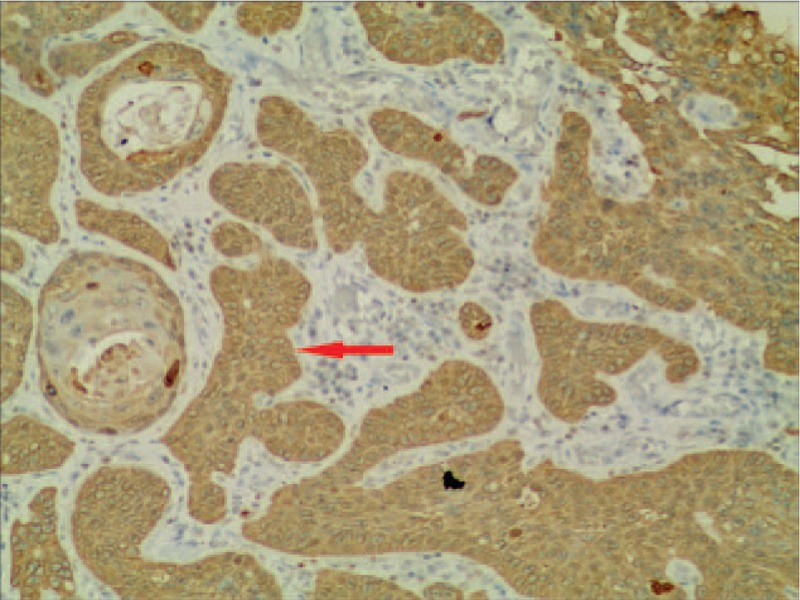
Section of tumor tissue stained using the EnVision method, and the CK5/6 was positive (arrow) (EnVision method; magnification, 200×).

**Figure 5:**
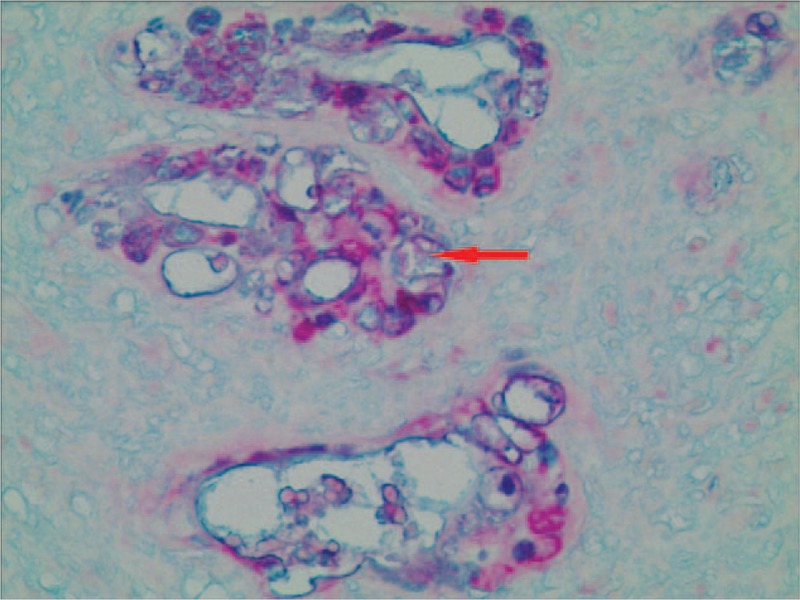
Mucus-secreting cells were typically visible in the tumor tissue (arrow) (Periodic Acid-Schiff staining, magnification, 200×).

Histopathology showed that most tumor tissue was composed of epidermoid cells that were arranged in nests. In addition, keratin pearl formation was identified. Columnar mucus-secreting cells and intermediate cells in small areas that formed tube-like structures with invasive growth were confirmed by hematoxylin and eosin (HE) staining (Fig. 2). Immunohistochemically, the tumor was found to be positive for p-63 (Fig. 3), and CK5/6 (Fig. 4), using the EnVision system (Agilent, Santa Clara, CA) to identify epidermoid cells. Mucus-secreting cells were identified using Periodic Acid-Schiff staining (Fig. 5). Based on immunohistochemistry results, the tumor was diagnosed as a poorly differentiated esophageal MEC. According to the esophageal cancer TNM (tumor, node, metastasis) staging system (American Joint Committee on Cancer/International Union for Cancer Control staging system, 2010), the pathological stage was IIIb: T3N1bM0. The tumor dimensions were 45 × 40 × 15 mm. The tumor invaded the outer membrane of the esophagus. All resection margins were negative for tumor involvement, and 3 of 12 lymph nodes from the upper abdominal and mediastinum showed signs of lymphatic metastasis.

### 2.5 Postoperative course

One month after surgery, radiotherapy combined with chemotherapy consisting of paclitaxel and cisplatin was provided. Four additional cycles of chemotherapy were administered; the regimen consisted of paclitaxel (200 mg/m^2^ on day 1 for 3 hours) plus cisplatin (30 mg/m^2^ per day for 3 days) repeated for 3 weeks. On the last day of the first chemotherapy cycle, radiotherapy against large chest irregular area, including the entire esophageal bed and the mediastina, was provided to the patient, beginning 30 minutes after the end of cisplatin infusion. The total dose for 95% of the planning target volume (PTV) was 50.4 Gy/28 fractions, and for 95% of the PTV for involved lymph nodes (PTVnd) was 56 Gy/28 fractions. No significant complications occurred after administration of adjuvant radio-chemotherapy. The patient was followed up with no evidence of recurrence for 17 months after surgery.

**Figure 6:**
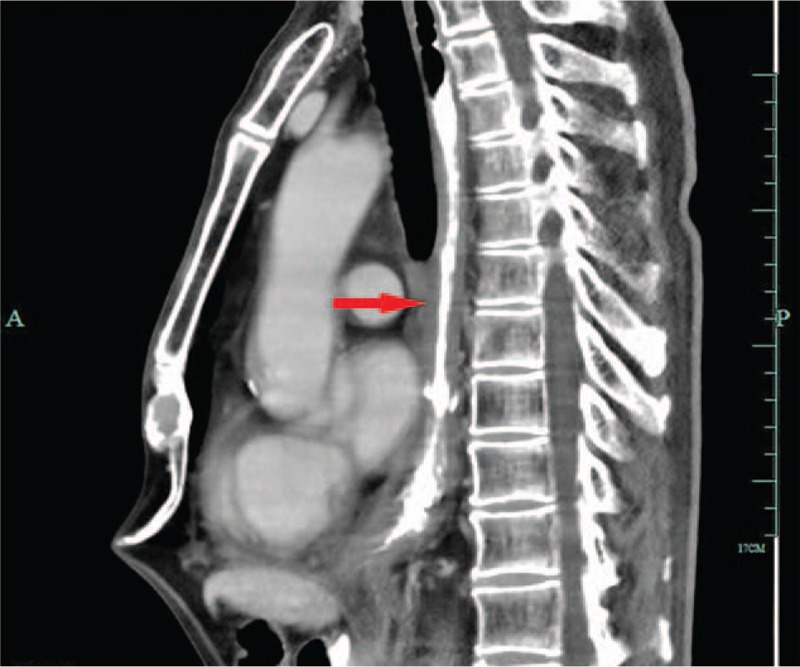
CT scanning at 17 months revealed that the esophageal wall was evenly thickened (arrow). CT = computed tomography.

**Figure 7:**
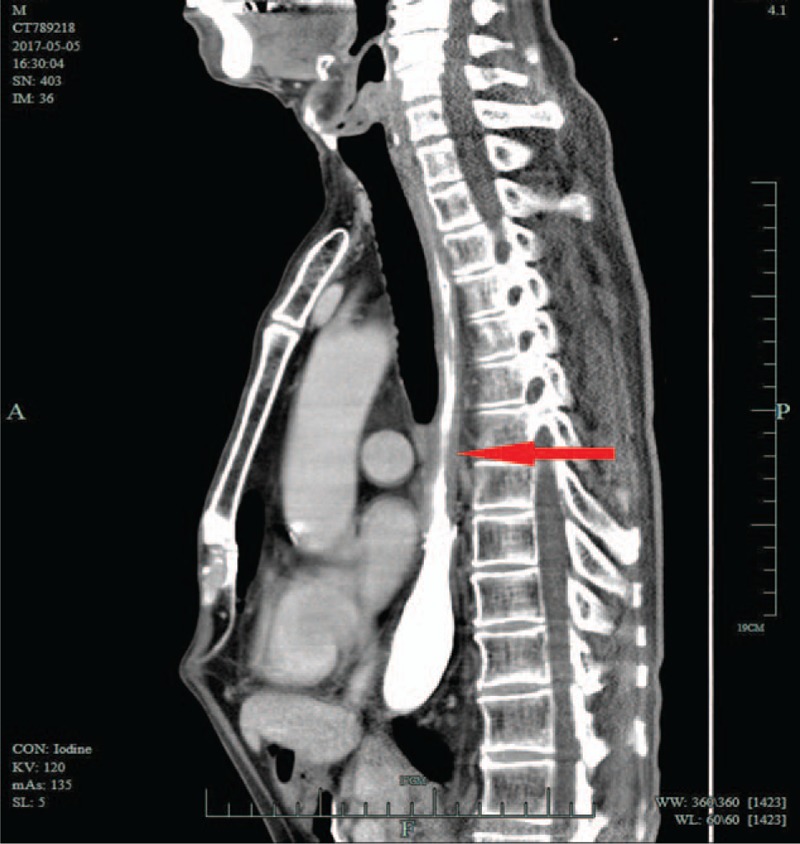
CT scanning at 31 months showed that the esophageal wall (arrow) remained thickened, similar to the findings from the esophageal CT scan 14 months prior (shown in Fig. 6). CT = computed tomography.

Seventeen months after surgery, an esophageal CT scan revealed that the esophageal wall was evenly thickened (Fig. 6). However, endoscopic assessment revealed no evidence of recurrence. Further esophageal CT scans at 19 and 31 months postsurgery also showed a thickened esophageal wall (Fig. 7); however, endoscopic assessment still revealed no esophageal stricture and no evidence of recurrence at 31 months after surgery. The patient was alive with no dysphagia and no evidence of recurrence for over 39 months.

## 3 Discussion

MEC is a malignant neoplasm that presents in the salivary glands, lacrimal and tracheobronchial glands, and rarely in the esophagus. Primary esophageal MEC accounts for 0.05% to 2.2% of all cases of primary esophageal cancer.^[1]^ As few reports on the biological behavior and treatment of esophageal MEC have been published, further investigation into the condition is required. Esophageal MEC mainly presents in the middle and lower thirds of the esophagus.^[2,3]^ Progressive dysphagia is the most common clinical symptom of esophageal MEC. Histologically, esophageal MEC is commonly characterized by mixture of epidermoid squamous cells, mucus-secreting cells, and intermediate cells. Due to the similar composition of epidermoid squamous cells to the squamous cell cancer in MEC tumor and the biopsy limitations, esophageal MEC is easily misdiagnosed as squamous cell cancer.^[3]^

Surgical resection is the primary treatment for esophageal MEC. However, even for patients who undergo radical resection, outcomes for this condition are poor, with a 5-year survival rate lower than the 5-year absolute survival rate for esophageal squamous cell carcinoma (ESCC).^[3]^ Lymph node metastasis and surgery have been identified as independent prognostic factors.^[3,4]^ Nobutoshi et al^[4]^ reported that neither chemotherapy nor radiotherapy were effective against esophageal MEC, and reported an overall median survival period of 10.8 months for esophageal MEC. In cases of unresectable or partially resectable tumors, or enhanced tumors, adjuvant treatment with chemotherapy or radiotherapy should be considered, even if the results were unsatisfactory. Turkyilmaz et al^[5]^ suggested that adjuvant chemotherapy and radiotherapy might prolong survival in some patients with esophageal MEC. Although neoadjuvant radio-chemotherapy has been used for the treatment of esophageal cancer patients and shown significant survival benefits,^[6]^ it has not been well studied in the treatment of esophageal MEC. Han et al^[7]^ showed that radiotherapy combined with chemotherapy consisting of paclitaxel and cisplatin is a safe and effective definitive treatment for locoregionally advanced ESCC. As the tumor in this case had a similar composition of squamous cells, we opted for chemotherapy with paclitaxel plus cisplatin during the radiation therapy for the radiosensitizing effect of cisplatin. Using the literature, we identified 30 minutes after cisplatin infusion as the optimal time to administer radiotherapy.^[8]^ The short-term effects were acceptable.

Following radio-chemotherapy, the patient showed a thickened esophageal wall. We performed endoscopic assessment and biopsy, but no evidence of recurrence of esophageal cancer was found. We suggest that this represents radiation esophagitis rather than recurrence of esophageal cancer. Radiation esophagitis can occur after radiotherapy, and is characterized by the presence of certain chronic changes including infiltration of fibroblasts and inflammatory cells into the muscular layer, submucosal edema, and fibrosis.^[9]^ These changes can lead to stenosis of the esophagus. Moreover, esophageal injury can develop after radiotherapy, and the typical esophageal lesion develops into esophageal stenosis after approximately 3 to 8 months of radiotherapy, which can lead to dysphagia.^[10]^ The incidence of esophageal stricture after radical radiotherapy is about 3%.^[10]^ Roeder et al^[11]^ believed that the occurrence of esophageal stenosis was significantly correlated with the radiation dose, and that the higher the radiation dose, the more fibrosis in the esophagus. In this case, the patient's esophageal wall was evenly thickened, but no obvious clinical symptoms nor evidence of recurrence were found in several follow-up visits. This indicates that the degree of radiation esophagitis was relatively mild. However, the specific identification of radiation esophagitis or tumor recurrence of this patient needs further follow-up and more biopsies.

According to some reports, the epidermal growth factor receptor (EGFR) is frequently overexpressed in salivary gland or pulmonary MEC, but few cases were positive for EGFR mutations. Tyrosine kinase inhibitor (TKI) therapy was reported with partial response in cases both with and without EGFR mutation for pulmonary MEC.^[12,13]^ Despite this, the role of TKI therapy in MEC remains unclear, especially in those patients who had a response to TKI therapy but had no activating EGFR mutations in their tumors. Currently, there are limited reports on targeted therapies for esophageal MEC. Although many researchers believe that targeted therapy might provide better benefits to patients, further investigation is needed.

## 4 Conclusion

The current study presents a rare case of esophageal MEC in a 59-year-old man which was successfully treated by surgical resection. Although the general prognosis of esophageal MEC is poor, comprehensive treatment consisting of surgery and radio-chemotherapy was effective in this case. To date, there is little evidence of effective treatment for esophageal MEC, nor guidelines for its treatment. This case and other reports suggest that adjuvant treatment is necessary for esophageal MEC. Radio-chemotherapy is a potential treatment option, with acceptable short-term effects.

## Author contributions

**Conceptualization:** Chunhui Zheng, Xiaomei Chen, Fangbiao Zhang, Xiangyan Zhang.

**Data curation:** Chunhui Zheng, Xiaomei Chen, Fangbiao Zhang, Liping Yan.

**Formal analysis:** Chunhui Zheng, Xiaomei Chen, Fangbiao Zhang, Xiangyan Zhang.

**Funding acquisition:** Chunhui Zheng.

**Investigation:** Chunhui Zheng, Xiaomei Chen, Fangbiao Zhang, Xiangyan Zhang.

**Methodology:** Chunhui Zheng, Fangbiao Zhang.

**Project administration:** Chunhui Zheng, Xiangyan Zhang.

**Resources:** Chunhui Zheng, Xiaomei Chen, Fangbiao Zhang.

**Software:** Chunhui Zheng.

**Supervision:** Chunhui Zheng.

**Validation:** Chunhui Zheng.

**Visualization:** Chunhui Zheng.

**Writing – original draft:** Chunhui Zheng.

**Writing – review and editing:** Chunhui Zheng.
